# The Benefits of Breastfeeding Still Outweigh the Risks of COVID-19 Transmission

**DOI:** 10.3389/fmed.2021.703950

**Published:** 2021-09-08

**Authors:** Fuxing Lou, Hongbo Qin, Shiting He, Maochen Li, Xiaoping An, Lihua Song, Yigang Tong, Huahao Fan

**Affiliations:** Beijing Advanced Innovation Center for Soft Matter Science and Engineering, College of Life Science and Technology, Beijing University of Chemical Technology, Beijing, China

**Keywords:** human breast milk, breastfeeding, COVID-19, ACE2, antiviral properties, SARS-CoV-2

## Introduction

The coronavirus disease 2019 (COVID-19) caused by severe acute respiratory syndrome coronavirus 2 (SARS-CoV-2) first broke out in Wuhan, China in December 2019, then swept the world, and was defined as a Public Health Emergency of International Concern (PHEIC) by World Health Organization (WHO) on 30 January 2020. As of July 21, 2021, 191,358,882 people have been infected, including 4,104,937 deaths (https://coronavirus.jhu.edu/map.html). The COVID-19 pandemic has seriously affected all patients, including lactating mothers.

The detection of severe acute respiratory syndrome coronavirus 2 (SARS-CoV-2) RNA in breast milk has raised concerns about mother-to-child transmission via breastfeeding (1–7). According to a recent coronavirus disease (COVID-19) survey of 1,344 hospitals (July 15-August 20, 2020) conducted by the United States Centers for Disease Control and Prevention, approximately two-third of the hospitals supported direct breastfeeding with caution, for mothers with suspected or confirmed COVID-19 (8). The consensus of Chinese experts suggests that mothers with COVID-19 and their infants should be routinely isolated, and breastfeeding should be stopped (9). Meanwhile, other guidelines suggest that mothers and their newborns should not be separated (10). With the controversial recommendations concerning breastfeeding of newborns born to mothers with suspected or confirmed COVID-19, several studies have been conducted in this regard. Considering the overall safety, it is essential to maintain strict hygiene during breastfeeding. This study aimed to review all such studies and offer suggestions on breastfeeding.

### SARS-CoV-2 RNA Detection in Breast Milk Is Not Equal to Infectivity

SARS-CoV-2 is continuously positive in respiratory droplets, feces, and breast milk. To date, no infectious virus particles have been isolated from breast milk (1, 4–7), and there have been no cases of transmission of infectious virus particles to infants through breast milk. In a study of 18 women infected with SARS-CoV-2 published in JAMA, none of them infected their children through breastfeeding (5). In another study from the World Health Organization (WHO), breast milk samples provided by 46 women infected with SARS-CoV-2 were tested, 43 were RNA-negative, and 3 were RNA-positive. One of the RNA-positive cases was a newborn with no breastfeeding history (7). Additionally, preterm infants of mothers infected with SARS-CoV-2 did not get infected even after being breastfed, which suggests that mothers infected with SARS-CoV-2 can safely breastfeed even their preterm infants (11). Notably, several infants with COVID-19 had received SARS-CoV-2-negative breast milk, including two newborns were exclusively breastfed. This suggests that the infants may have become infected with SARS-CoV-2 through close contact with infected family members, which seems plausible (12). In fact, there are no reports on transmission of SARS-CoV and Middle East respiratory syndrome coronavirus (MERS-CoV) via breast milk (13, 14). Moreover, it seems impossible to culture infectious viruses from RNA-positive samples of breast milk, and the replication-competent virus was detected in none of the breast milk samples provided by the infected women, including those that tested positive for viral RNA. Evidence suggests that the SARS-CoV-2 virus particles detected in breast milk may not be infectious, and breastfeeding may not be the cause of infection in infants (4, 5, 15, 16). Therefore, viral RNA detection is not equal to infectivity, clinical samples positive for SARS-CoV-2 RNA should be tested for live viruses, and it is the basis for understanding infectivity (16, 17).

### COVID-19 Infection Is Uncommon and Rarely Symptomatic in Newborns

Evidence shows that infants infected with COVID-19 generally have mild symptoms, and the biological basis for this phenomenon is unclear (7, 18–21). The susceptibility of persons aged 15–64 years is higher than that of children aged 0–14 years. In addition, children usually have milder symptoms and a lower mortality, which has also been reported in infections with SARS-CoV and MERS-CoV (22). A survey found that only 1% of 70,000 patients in China were aged below 10 years (23). Additionally, pediatric patients with SARS-CoV or SARS-CoV-2 usually have a better prognosis than their adult counterparts (18). In a previous study of 11 children with COVID-19, none of the children had severe symptoms (24, 25). COVID-19 in newborns is uncommon, rarely symptomatic, and the rate of infection is lower when the baby is delivered naturally (7), which may indicate that vertical perinatal transmission does not occur. IgM and IgG levels are persistently high in uninfected newborns born to infected mothers (26), which may be explained by passive immunity during pregnancy.

### The Expression Level of Angiotensin Converting Enzyme 2 in Mammary Epithelial Cells Is Extremely Low

Angiotensin converting enzyme 2 (ACE2) is a functional receptor of SARS-CoV (27), SARS-CoV-2 (28), and SARS-CoV-2-related pangolin coronavirus (GX_P2V) (29). SARS-CoV-2 viral RNA could not be detected in most breast milk samples of infected mothers, which may be possibly due to low levels of ACE2 expression in the breasts (30–32). ACE2 expression in the female reproductive system, including the mammary glands, is extremely low, suggesting a low possibility of high levels of infectious SARS-CoV-2 in breast milk (10). In fact, ACE2 needs to be co-expressed with the protease, TMPRSS2 or CTSB/L, to activate the S protein to promote the entry of SARS-CoV-2 into host cells. However, no ovarian cell co-expressed ACE2 with TMPRRS2, CTSB, and/or CTSL, and only 5% of mammary gland cells expressed ACE2 (11), suggesting that there is no risk of vertical transmission of SARS-COV-2 from mother-to-child via breast milk. However, other possible routes of transmission of infection to infants cannot be neglected. These include through breastfeeding-related body fluids such as blood, sweat, and respiratory droplets; droplet transmission caused by close contact; skin-to-skin exposure; and gas transmission (5, 21). COVID-19 is a respiratory disease, and there is still no evidence for transmission of SARS-CoV-2 via food, including breast milk.

### Breast Milk Provides Energy, Nutrition, Growth Factors, and Oligosaccharides to Newborns

The benefits of breastfeeding greatly outweigh the potential risks of COVID-19 transmission, which explains why WHO recommends that patients with confirmed/suspected COVID-19 should continue breastfeeding (10). Breastfeeding is beneficial to mothers and infants: it helps to reduce the risk of breast cancer and ovarian cancer for mothers and significantly reduces neonatal mortality (10). Apart from providing energy and nutrition, breast milk contains growth factors, oligosaccharides, and immunoglobulins, which are particularly important for the development and protection of newborns (33). Furthermore, studies have shown that the constituents of the breast milk of patients with COVID-19 are not significantly different from those of healthy people (34), unlike formula milk that lacks many important constituents found in human milk. COVID-19 may cause severe cytokine storms that lead to exaggerated inflammatory responses in patients, which can be effectively regulated by milk and its immunoregulatory factors in early life (35).

### Breast Milk Contains Components With Antiviral Properties

**Figure 1:**
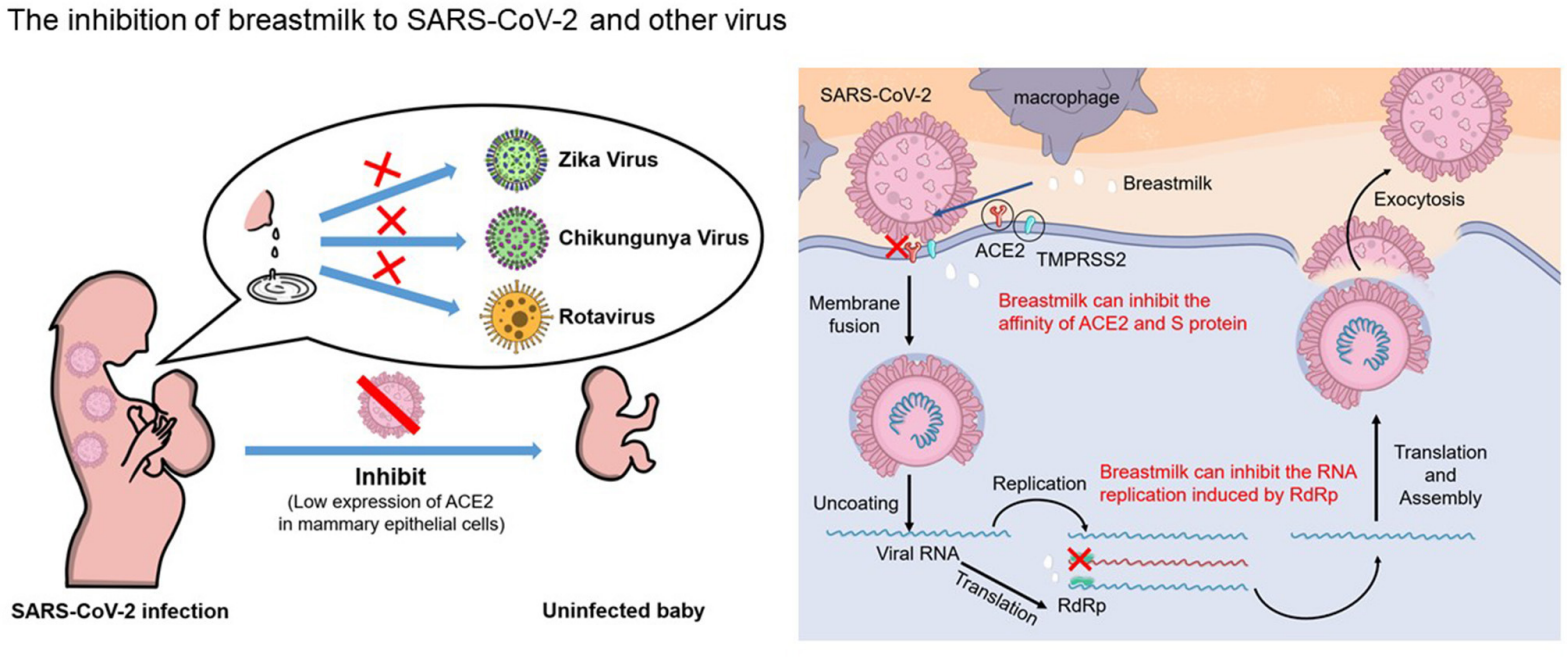
Human breastfeeding should be encouraged for the mothers who are suspected or confirmed with COVID-19. Breast milk provide protection against several pathogens including Zika virus, chikungunya virus and rotavirus; for SARS-CoV-2, breast milk not only block the binding between ACE2 and spike protein, but also potently inhibit RdRp activity of SARS-CoV-2. The antiviral effects of breast milk and low levels of ACE2 expression in the breasts could explain that breast-fed newborns are less likely to be infected with virus from their COVID-19 mothers through breast milk.

Breast milk has antiviral properties (36–39), and it has recently been proven to have an anti-SARS-CoV-2 function (40, 41) (Figure 1). In a previous study, when five different strains of SARS-CoV-2 were mixed with breast milk at room temperature for 30 min, the virus titer decreased by 40.9 ~92.8%, indicating that breast milk had an antiviral activity (40). Lactoferrin (42), linoleic acid (43–45), and IgA (46–48) have antiviral functions. Lactoferrin, the main whey protein in human milk, has a wide range of antimicrobial and immunomodulatory functions and plays an important role in regulating the infantile immune system (35). It confers protection against several pathogens, including papillomavirus (36), human immunodeficiency virus (37), rotavirus (38), chikungunya virus (39), and zika virus (39). It may also inhibit SARS-CoV-2 by binding to some of the receptors required for viral entry, such as ACE2 and HSPGs (42, 49). An *in vitro* study confirmed that lactoferrin inhibits SARS-CoV-2 infection and replication in Caco-2 cells (50). In addition, an *in vivo* study confirmed that lactoferrin accelerates SARS-CoV-2 RNA-negative conversion in patients with COVID-19 (51).

Recent studies have shown a high abundance of oleic and linoleic acids in human breast milk, and bound linoleic acid stabilizes a locked SARS-CoV-2 spike protein conformation, resulting in reduced ACE2 interaction *in vitro*. A synergy of remdesivir and linoleic acids are believed to suppress SARS-CoV-2 replication in human epithelial cells (44). Moreover, some reports suggest that IgA and IgG of SARS-CoV-2 can be detected in breast milk. IgA and IgG antibodies were detected in 12/15 breast milk samples from patients with COVID-19 (46). IgA and IgG antibodies in the breast milk of patients with COVID-19 effectively neutralize the infectivity of SARS-CoV-2 (47). Therefore, breastfeeding may lead to passive immunity, which is protective. In our recent study, whey protein samples from human breast milk stored in 2017 could not only inhibit the binding of spike protein and ACE2 in a dose-dependent manner, but could also strongly inhibit the activity of RNA-dependent RNA polymerase of SARS-CoV-2, which are important for virus entry and replication, respectively (41). This may also be an important reason why infectious virus particles have not been isolated from breast milk.

### The Effects of SARS-CoV-2 Drug Therapy on Breastfeeding

Considering that infants may obtain COVID-19 medications through breastfeeding from their mothers, excretion in breast milk of various medications including anakinra, remdesivir, hydroxylchloroquine, chloroquine, ribavirin, favipiravir, and dexamethasone used in COVID-19 therapy are summarized. Anakinra is a normal component in breast milk, its content in the colostrum of healthy mothers is 672 ± 202 ng/ L, and 316 ± 70 ng/ L in mature milk (52), and infants breast-fed by mothers who received 100 mg of anakinra daily did not experience adverse reactions (53–56). Remdesivir, the only drug authorized by the US Food and Drug Administration for emergency use of COVID-19 therapy, and the information of its excretion in breast milk is not available. Moreover, there is no report on the adverse effect of infants breastfeeding from mothers with remdesivir treatment, no adverse reactions was found in the infants after intravenous remdesivir injection for Ebola therapy (57), suggesting the benefits of breastfeeding outweigh the potential risks of remdesivir. The amount of hydroxylchloroquine excreted from breast milk is low, and its amount in the breast milk was 3.2 ug after an oral regimen of 800 mg for 24 h (58), and infants receive no more than 0.2mg/kg of hydroxylchloroquine through breastfeeding from mothers who had 200 mg of hydroxychloroquine daily (59). In addition, 130 breastfed infants whose mothers received hydroxychloroquine treatment appeared to have normal development (60). Breastfeeding for mothers during hydroxychloroquine treatment are also recommended (61, 62). Similarly, the excretion of chloroquine in breast milk is also in a safety range for the infants. The average level of chloroquine in breast milk of 6 mothers who received 300mg chloroquine daily was 3.97 mg/l, and it was estimated that breastfed babies received only 0.55% of the total dose of the mother's daily dose (63). United Kingdom malaria treatment guidelines recommends that weekly 500 mg chloroquine in mothers is acceptable for infants during breastfeeding. The excretion in breast milk of ribavirin is unclear, however, as a drug can be given directly to infants, obtaining ribavirin through breastfeeding might be safe for infants (https://www.ncbi.nlm.nih.gov/books/NBK500613/).

Currently, the information on the safety of favipiravir targeting SARS-CoV-2 RdRp during breastfeeding is not available (https://www.ncbi.nlm.nih.gov/books/NBK556878/). However, considering favipiravir may exist in breast milk as a small molecule, and cause adverse reactions such as liver enzyme abnormalities (64, 65), it is necessary to monitor its exudation in breast milk. Similarly, there is no information about dexamethasone transmission to infants through breast milk. However, some studies have found that infants exposed to topical dexamethasone will experience severe hypertension, decreased growth and electrolyte abnormalities (66). High level excretion of dexamethasone into breast milk is possible (https://www.ncbi.nlm.nih.gov/books/NBK501758/), considering its potential adverse effects on infants, it is necessary to monitor its excretion in breast milk.

In a word, it is safe for the infants breastfed from COVID-19 mothers receiving medications therapy including anakinra, remdesivir, hydroxylchloroquine, chloroquine, ribavirin, and the usage of favipiravir and dexamethasone for COVID-19 mothers during breastfeeding should be continually monitored.

## Discussion

Overall, human breastfeeding should be encouraged, and for a mother with confirmed or suspected COVID-19 who is unable to breastfeed, expressed breastmilk is the best alternative to direct breastfeeding of a newborn or young infant (10). Pasteurization effectively inactivates SARS-CoV-2-spiked breast milk (40, 67). Considering that SARS-CoV-2 is sensitive to heat (68), Unger et al. used the Holder method (62.5°C, 30 min) to pasteurize breast milk with SARS-CoV-2 and found that it could completely inactivate the virus (67). Therefore, pasteurization can ensure the safety of breastfeeding to a certain extent. However, some studies have shown that pasteurization reduces the inhibitory effect of IgA on SARS-CoV-2 (69) without reducing IgA levels in breast milk significantly but impairing the neutralizing ability of IgA (47). Therefore, pasteurization cannot be used as a benign intervention because of its effect on immunoactive components of breast milk (70).

Therefore, the dangers of stopping breastfeeding greatly outweighs the potential risk of COVID-19 transmission, and human breast feeding should be encouraged for mothers with suspected or confirmed COVID-19. Considering other possible transmission routes of SARS-COV-2, measures should be taken to maintain strict hygiene during breastfeeding.

## Author Contributions

HF, LS, and YT designed the research. HF and FL drafted the manuscript. HF, FL, HQ, SH, and ML collected the materials and read the literatures. All the authors were involved in the manuscript discussion.

## Funding

This research was supported by National Key Research and Development Program of China (Nos. 2020YFA0712100, 2018YFA0903000, 2020YFC2005405, and 2020YFC0840805), Key Project of Beijing University of Chemical Technology (No. XK1803-06), Funds for First-class Discipline Construction (No. XK1805), Inner Mongolia Key Research and Development Program (No. 2019ZD006), National Natural Science Foundation of China (Nos. 81672001 and 81621005), NSFC-MFST project (China-Mongolia) (No. 31961143024), and Fundamental Research Funds for Central Universities (Nos. BUCTRC201917 and BUCTZY2022). H&H Global Research and Technology Center (grant No. H2021028).

## Conflict of Interest

The authors declare that the research was conducted in the absence of any commercial or financial relationships that could be construed as a potential conflict of interest.

## Publisher's Note

All claims expressed in this article are solely those of the authors and do not necessarily represent those of their affiliated organizations, or those of the publisher, the editors and the reviewers. Any product that may be evaluated in this article, or claim that may be made by its manufacturer, is not guaranteed or endorsed by the publisher.
